# *BRCA2* Reversion Mutation after Neoadjuvant Dose-Dense EC and Dose-Dense Paclitaxel in Triple-Negative Breast Cancer: A Case Report and Literature Review

**DOI:** 10.70352/scrj.cr.25-0632

**Published:** 2026-03-18

**Authors:** Hajime Hikino, Asa Otani, Yoshinari Makino

**Affiliations:** Department of Breast Surgery, Matsue Red Cross Hospital, Matsue, Shimane, Japan

**Keywords:** *BRCA2*, reversion mutation, triple-negative breast cancer, chemotherapy resistance, homologous recombination

## Abstract

**INTRODUCTION:**

*BRCA* reversion mutations are known mechanisms of acquired resistance to poly (ADP-ribose) polymerase inhibitors (PARPis) and platinum agents. However, their clinical emergence without such therapies is rarely reported.

**CASE PRESENTATION:**

We describe a 44-year-old woman with early-stage triple-negative breast cancer carrying a germline *BRCA2* mutation who developed a *BRCA2* reversion mutation after neoadjuvant dose-dense epirubicin and cyclophosphamide (EC) followed by dose-dense paclitaxel, without prior PARPi or platinum exposure. She underwent modified radical mastectomy and achieved a good pathological response; however, she developed early systemic recurrence, including leptomeningeal metastasis, 7 months postoperatively. Comprehensive genomic profiling of the residual breast tumor revealed a *BRCA2* reversion mutation (allele frequency: 6.7%) that restored the open reading frame. We speculate that the genomic instability in the tumor may have induced a spontaneous reversion mutation early in the disease course.

**CONCLUSIONS:**

This case suggests that a *BRCA2* reversion mutation can arise early, even before PARPi or platinum exposure. Serial monitoring of *BRCA* status may help predict therapeutic resistance in patients with germline *BRCA*-mutated breast cancer.

## Abbreviations

cfDNA
: cell-free DNA
CGP
: comprehensive genomic profiling
EC
: epirubicin and cyclophosphamide
*gBRCA*
: germline BRCA
PARPi
: poly (ADP-ribose) polymerase inhibitor
TNBC
: triple-negative breast cancer

## INTRODUCTION

In Japan, approximately 4.2% of unselected breast cancers are reported to possess germline *BRCA1* or *BRCA2* mutations.^1)^ These mutations impair homologous recombination repair, rendering tumors sensitive to PARPis and platinum agents through synthetic lethality.^2)^ However, resistance can develop due to secondary reversion mutations that restore *BRCA* function.^3,4)^

Reversion mutations typically arise after exposure to PARPi or platinum agents. Here, we present a rare case of a *gBRCA2*-mutated TNBC patient who developed a *BRCA2* reversion mutation after neoadjuvant chemotherapy with dose-dense EC and dose-dense paclitaxel, without exposure to PARPi or platinum agents. This case highlights the possibility of spontaneous reversion mutations early in the clinical course and their potential clinical significance.

## CASE PRESENTATION

**Fig. 1:**
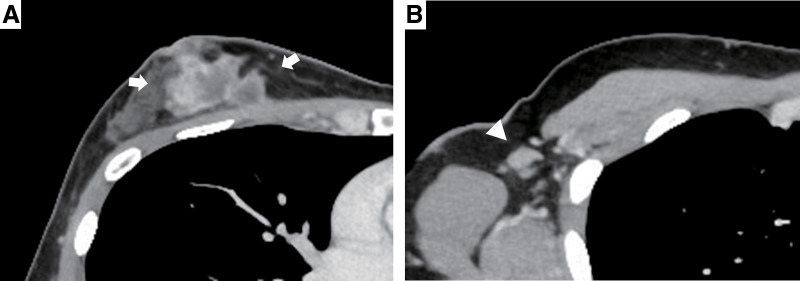
Contrast-enhanced CT images at presentation revealed a 4.3 × 4.3-cm tumor in the upper-inner quadrant of the right breast (**A**, arrows), with enlarged axillary lymph nodes (**B**, arrowhead).

**Fig. 2:**
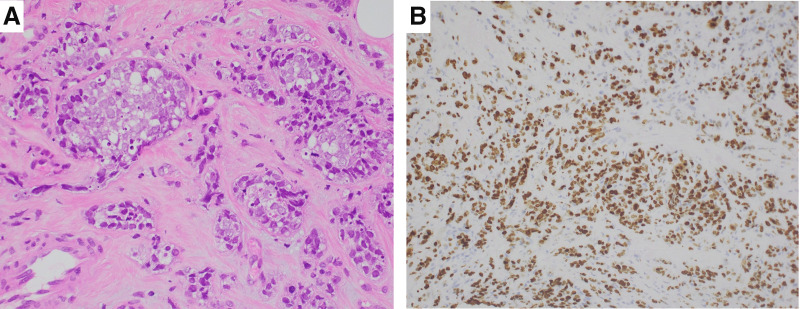
Photomicrographs of the core needle biopsy specimen. (**A**) Tumor cells showed marked nuclear atypia and mitoses infiltrating surrounding tissue in alveolar and tubular patterns (H&E stain, ×400). (**B**) MIB-1 labeling index was 90% (×200). H&E, hematoxylin and eosin; MIB-1, molecular immunology Borstel-1

A 44-year-old woman presented with a 4.3 × 4.3-cm mass in the upper-inner quadrant of the right breast. She had no significant medical history but reported a family history of ovarian cancer (paternal grandmother) and pancreatic cancer (paternal uncle). A CT revealed a contrast-enhancing breast mass with 3 axillary lymphadenopathies (**Fig. 1**). Core needle biopsy showed high-grade invasive ductal carcinoma, negative for estrogen receptor, progesterone receptor, and HER2, with Ki-67 expression of 90% (**Fig. 2**). Genetic testing (BRACAnalysis; Myriad Genetics, Salt Lake City, UT, USA) identified a pathogenic *gBRCA2* c.6014_6017del (p.D2005Vfs*34) mutation. The clinical stage was cT2N1M0 (Stage IIB).

She received neoadjuvant chemotherapy using 4 cycles of dose-dense EC (epirubicin 90 mg/m^2^ and cyclophosphamide 600 mg/m^2^ every 2 weeks), followed by 4 cycles of dose-dense paclitaxel (175 mg/m^2^ every 2 weeks), with pegfilgrastim 3.6 mg per cycle. She then underwent a right modified radical mastectomy and contralateral risk-reducing mastectomy. Pathology revealed a 7-mm residual invasive carcinoma (triple-negative, Ki-67 73%). PD-L1 was negative on immunohistochemistry (PD-L1 antibody clone SP142; Ventana Medical Systems, Tucson, AZ, USA; PD-L1 antibody clone 22C3 pharmDx; Dako, Glostrup, Denmark). No nodal involvement was noted, and pathological staging was ypT1bN0M0 (ypStage IA). The left breast was free of malignancy.

**Fig. 3:**
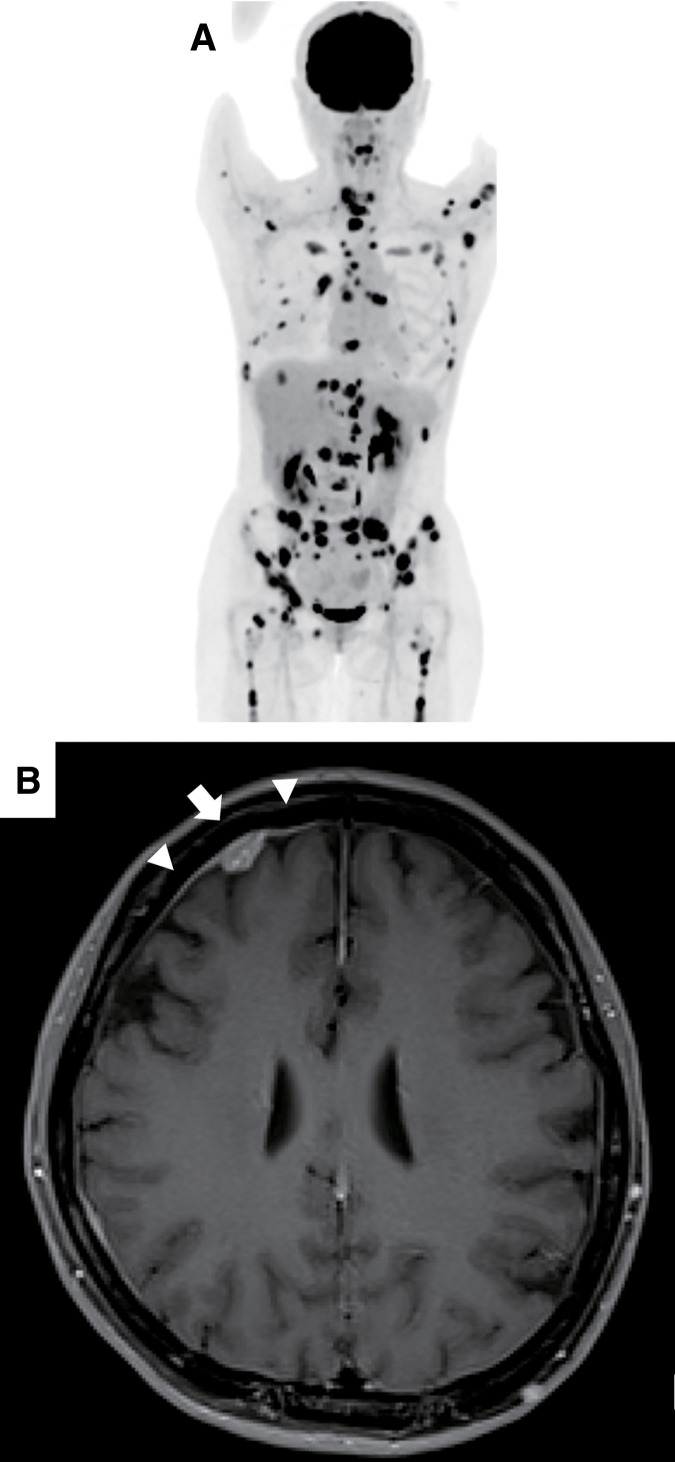
(**A**) ^18^F-FDG PET/CT showed abnormal uptakes in bone, liver, lung, and distant lymph nodes, indicating multiple systemic recurrences. (**B**) Gadolinium-enhanced T1-weighted MRI of the brain revealed a nodular enhancing lesion (arrow) and leptomeningeal involvement (arrowheads) in the right frontal lobe, consistent with carcinomatous meningitis.

Adjuvant therapy included post-mastectomy radiotherapy (50 Gy in 25 fractions) to the right chest wall and supraclavicular area, and capecitabine (1250 mg/m^2^ twice daily on days 1–14 every 3 weeks). At 7 months post-surgery, during the fifth cycle of capecitabine, she developed widespread metastases to bone, liver, lung, lymph nodes, and leptomeninges, confirmed by PET (**Fig. 3A**) and brain/spine MRI (**Fig. 3B**). Cerebrospinal fluid cytology confirmed leptomeningeal involvement. Rapid clinical deterioration precluded PARPi therapy; weekly paclitaxel (90 mg/m^2^) plus biweekly bevacizumab (10 mg/kg) and zoledronic acid were initiated.

**Fig. 4:**
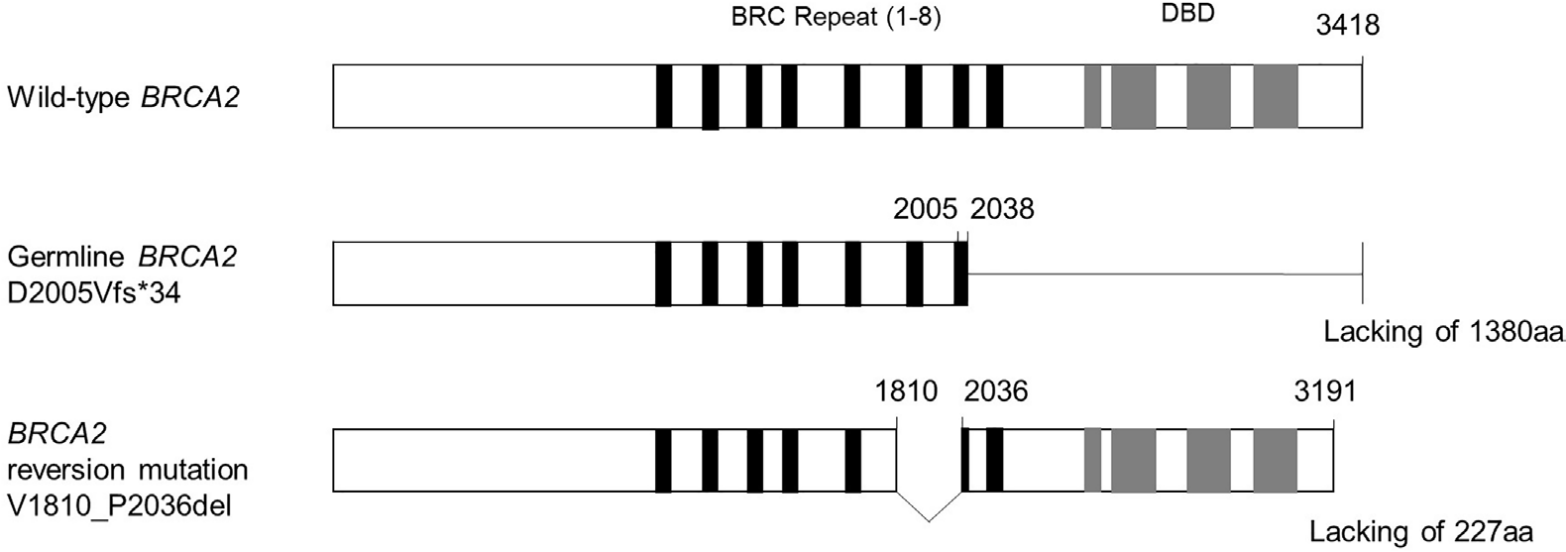
Illustration of the wild-type *BRCA2*, the germline frameshift mutation, and the *BRCA2* reversion mutation. The reversion mutation encompassed the frameshift site and restored an open reading frame, resulting in a protein of 3191 amino acids lacking 227 residues. aa, amino acid; DBD, DNA-binding domain

CGP (FoundationONE; Foundation Medicine, Boston, MA, USA) of the residual primary tumor, with an estimated tumor content of approximately 20%, identified the original *BRCA2* frameshift mutation (allele frequency: 44.8%) and a *BRCA2* reversion mutation c.5429_6109del681 (p.V1810_P2036del; allele frequency: 6.7%) that restored the reading frame (**Fig. 4**). Additional mutations included *PIK3CA* c.1637A>G (p.Q546R), *CTNNA1* c.1256_1265del (p.Y419fs*9), and low tumor mutational burden.

The patient’s general condition was significantly compromised, leading to her death a month later (11 months after surgery).

## DISCUSSION

We present a rare case of a *BRCA2* reversion mutation occurring after neoadjuvant chemotherapy with dose-dense EC and dose-dense paclitaxel, without prior PARPi or platinum agents. In this case, the original germline *BRCA2* mutation was a frameshift deletion at positions c.6014–6017, resulting in the loss of *BRCA2* function. After neoadjuvant chemotherapy, a secondary large in-frame deletion (c.5429–6109del) was identified, which encompassed and removed the region containing the original frameshift mutation. As a result, the open reading frame of *BRCA2* was restored, potentially allowing partial recovery of homologous recombination function. This mechanism is well documented in PARPi and platinum resistance.^5,6)^ The variant allele frequency of the *BRCA2* reversion mutation was 6.7%. Given the estimated tumor content of approximately 20% in the analyzed tissue, this finding suggests that the reversion mutation was present in a minor to intermediate subclonal tumor population.

Although reversion mutations are known to occur under intensive therapeutic selection pressure from PARPi or platinum agents, the precise mechanism and prevalence of reversion mutations remain unclear.^5,7,8)^ We performed a literature search in the PubMed database using the words “*BRCA*,” “reversion,” “mutation,” and “secondary mutation”. We excluded data that did not describe the clinical course of the disease. A total of 171 cases were reported, with a predominance of *BRCA2* (121 cases) compared with *BRCA1* (50 cases). In *BRCA2*-mutated tumors, reversion events often involve large deletions within exon 11 encoding the BRC repeat domain, where partial *BRCA2* function may be retained. This characteristic may contribute to the higher frequency of reversion mutations observed in *BRCA2*.^5,9)^ However, the precise molecular mechanisms underlying this tendency remain to be fully elucidated. By cancer type, 60 were ovarian, 59 prostate, 35 breast, 14 pancreatic, 1 gallbladder, 1 esophageal, and 1 non-small cell lung cancer. Only 5 of 171 cases (2.9%) occurred without prior PARPi or platinum exposure, with 4 detected by cfDNA (**Table 1**).^7,10–12)^ In these cases, patients had typically received intensive chemotherapy including DNA cross-linking agents such as cyclophosphamide, which may exert similar selection pressure.^13)^

**Table 1:** Reported cases of *BRCA* reversion mutations without prior exposure to PARPis or platinum-based therapy

Author	Year	Cancer type	Status	Deleterious mutation	CGP specimen	Prior systemic therapies
Nakamura et al.^7)^	2024	Pancreas	Metastasis	*gBRCA2*	Liquid	Non-platinum-based chemotherapy
Carneiro et al.^10)^	2018	Prostate	Metastasis	*gBRCA2*	Liquid	Taxane, mitoxantrone, radium-223
Vidula et al.^11)^	2020	Breast	Metastasis	*gBRCA2*	Liquid	ET, capecitabine, everolimus, paclitaxel, eribulin, gemcitabine, mitoxantrone, vinorelbine
		Prostate	Metastasis	*gBRCA2*	Liquid	ADT, docetaxel, enzalutamide, abiraterone acetate, cabazitaxel, mitoxantrone
Murciano-Goroff et al.^12)^	2022	Breast	Primary	*gBRCA1*	Tumor	Doxorubicin, cyclophosphamide, docetaxel, paclitaxel
Present case	2025	Breast	Primary	*gBRCA2*	Tumor	Epirubicin, cyclophosphamide, paclitaxel

ADT, androgen deprivation therapy; CGP, comprehensive genomic profiling; ET, endocrine therapy; *gBRCA*, germline *BRCA*; PARPi, poly (ADP-ribose) polymerase

Reversion mutations are generally rare stochastic events that usually arise in advanced cancers with high genomic instability.^14)^ However, our case is unique in that the mutation was detected in residual tumor after neoadjuvant therapy for early-stage disease, suggesting that a small clone harboring the reversion mutation may have pre-existed or been induced early during chemotherapy. Given the elevated genomic instability associated with homologous recombination deficiency in *gBRCA*-mutated TNBC,^15)^ spontaneous reversion may occur more readily in this context. Notably, the patient experienced rapid recurrence and died of the disease, but whether *BRCA* reversion mutations directly increase intrinsic tumor aggressiveness remains uncertain. Recognition of this phenomenon is critical for treatment decisions and for developing strategies to overcome therapeutic resistance.

This study has limitations. We did not use PARPi in either the adjuvant or metastatic setting. Adjuvant olaparib has shown significant overall survival benefit in TNBC patients with *gBRCA* mutations and residual disease after intensive neoadjuvant therapy.^16)^ At the time of treatment, adjuvant PARPi was not yet approved in Japan for *gBRCA*-mutated breast cancer. In the metastatic setting, the presence of a reversion mutation raised concern for intrinsic PARPi resistance. Furthermore, the patient’s rapid decline precluded oral targeted therapy.

## CONCLUSIONS

*BRCA2* reversion mutations can arise early in the course of *gBRCA*-mutated breast cancer, even without PARPi or platinum exposure. Serial monitoring using liquid biopsies may help detect these events, guide treatment selection, and predict resistance. Further studies are needed to clarify the incidence, timing, and mechanisms of reversion mutations in breast cancer.

## DECLARATIONS

### Availability of data and materials

The data that support the findings of this study are available from the corresponding author upon reasonable request.
